# Perinatal Bisphenol A Exposure Increases Estrogen Sensitivity of the Mammary Gland in Diverse Mouse Strains

**DOI:** 10.1289/ehp.9640

**Published:** 2007-01-17

**Authors:** Perinaaz R. Wadia, Laura N. Vandenberg, Cheryl M. Schaeberle, Beverly S. Rubin, Carlos Sonnenschein, Ana M. Soto

**Affiliations:** Department of Anatomy and Cellular Biology, Tufts University School of Medicine, Boston, Massachusetts, USA

**Keywords:** BPA, C57Bl6, CD-1, estradiol, estrogen bioassay, mammary gland bioassay, mouse strains, nonmonotonic response, uterotropic assay

## Abstract

**Background:**

Studies of low-dose effects of xenoestrogens have yielded conflicting results that may be attributed to differences in estrogen sensitivity between the rodent strains examined. Perinatal exposure of CD-1 mice to low doses of the xenoestrogen bisphenol A (BPA) alters peripubertal mammary gland development. Future studies to assess the role of estrogen receptors as mediators of BPA action require estrogen receptor knock-out mice that were generated on a C57Bl6 background. The sensitivity of the C57Bl6 strain to estradiol and BPA is unknown.

**Objectives:**

In the present study we examined whether the mammary glands of CD-1 and C57Bl6 mice exhibited similar responses to 17β-estradiol (E_2_) and whether perinatal exposure to BPA equally enhanced sensitivity of the mammary glands to E_2_ at puberty.

**Methods:**

Immature mice were ovariectomized and treated for 10 days with one of eight doses of E_2_. Morphological mammary gland parameters were examined to identify doses producing half-maximal effects. Mice were exposed perinatally to 0 or 250 ng BPA/kg body weight (bw)/day from gestational day 8 until postnatal day (PND) 2. On PND25, female offspring were ovariectomized and given an estrogen challenge of 0, 0.5, or 1 μg E_2_/kg bw/day for 10 days. Morphometric parameters of the mammary gland were compared between strains.

**Results:**

Both strains exhibited similar responses to E_2_. Perinatal BPA exposure altered responses to E_2_ at puberty for several parameters in both strains, although the effect in CD-1 was slightly more pronounced.

**Conclusion:**

Both mouse strains provide adequate models for the study of perinatal exposure to xenoestrogens.

Environmental exposure to hormonally active chemicals has coincided with an increase in the incidence in breast cancer (Davis et al. 1993), testicular cancer (Skakkebaek et al. 1998), and other endocrine-related diseases (Sharpe and Skakkebaek 1993). These outcomes are thought to result from extemporaneous exposure to synthetic estrogens during fetal development (Sharpe and Skakkebaek 1993) and have motivated the worldwide formation of government-sponsored committees to evaluate evidence for this hypothesis. For instance, in the United States, the U.S. Environmental Protection Agency (EPA) developed a screening program to test chemicals that may contaminate water and food to assess potential endocrine disruptor activity (Endocrine Disruptor Screening and Testing Advisory Committee 1998).

At the request of the U.S. EPA, the National Toxicology Program (NTP) convened a meeting to consider whether environmentally relevant doses of endocrine disruptors caused biological effects. In 2001, the NTP Low-Dose Peer Review Panel published a final report (NTP 2001), which stated that there was “credible evidence for low-dose effects” and suggested that different experimental animal strains may account for reports of both positive and negative effects for the same parameters. In this regard, Spearow et al. (1999) observed that rodent strains selected for high fecundity and rapid growth rates, such as CD-1 mice, are more estrogen resistant than the less fecund C57Bl6.

An additional controversy identified by the NTP was the shape of the dose–response curve, which was reported as nonmonotonic for some effects of prenatal xenoestrogen exposure. For example, prenatal methoxychlor exposure altered the response of the adult uterus to 17β-estradiol (E_2_); low doses increased uterine weight, and higher doses reduced it (Alworth et al. 2002). This type of nonmonotonic response was also observed for other end points with other estrogenic chemicals (Rubin et al. 2001; Vandenberg et al. 2006; vom Saal et al. 1997), arguing that low-dose effects cannot be deduced from effects observed at high doses.

Our research focuses on the effects of perinatal exposure to environmentally relevant levels of the xenoestrogen bisphenol A (BPA). In the the present study we examined strain sensitivity and the shape of the estrogen dose–response curve in the context of our ongoing work in the mouse mammary gland. In addition, we explored the effects of perinatal BPA exposure on subsequent estrogen sensitivity at puberty.

BPA, a compound used in the manufacture of polycarbonate plastics and epoxy resins, leaches from food and beverage containers (Biles et al. 1997; Brotons et al. 1994) and dental sealants and composites (Olea et al. 1996) under normal conditions of use (Markey et al. 2001b, 2003b). BPA levels have been measured in human urine (Calafat et al. 2005), serum (Takeuchi and Tsutsumi 2002), and maternal and fetal plasma, amniotic fluid, and placental tissue at birth (Ikezuki et al. 2002; Schonfelder et al. 2002). We chose to administer perinatally 0 or 250 ng BPA/kg body weight (bw)/day to mice. Based on data reported by Arakawa et al. (2004), we estimated that this level of BPA should fall within the range of reported human exposures.

In the mammary gland, perinatal exposure to BPA alters ductal invasion of the stroma at puberty and increases lateral branching and the number of terminal ends during adulthood (Markey et al. 2001a; Munoz de Toro et al. 2005). Although the mechanisms by which BPA induces developmental abnormalities in the mammary gland are unknown, it is plausible that estrogen receptors (ER), which are expressed in the fetal mammary gland, may mediate BPA-induced effects.

In the present study, we compared the response of the mammary glands to E_2_ in outbred CD-1 mice (the strain we have used previously) with inbred C57Bl6 mice, which have been used in numerous studies involving development of the mammary gland. Different levels of biological complexity within the mammary gland (i.e., tissue organization and gene expression) were examined. We also examined the effects of perinatal BPA exposure on the response to E_2_ at puberty in both strains.

## Materials and Methods

### Animals

CD-1 (Crl: CD-1; Charles River Laboratories, Wilmington, MA) and C57Bl6 (C57BL/6J; Jackson Laboratories, Bar Harbor, Maine) mice were housed in the Tufts-New England Medical facility under temperature and light controlled (14-hr light and 10-hr dark) conditions. Food (Rodent Diet 2018; Harlan Teklad, Madison, WI), cages, and bedding tested negligible for estrogenicity by the E-SCREEN assay (Soto et al. 1992); water was supplied in glass bottles. All experimental procedures were approved by the Tufts University–New England Medical Center Animal Research Committee. All animals were treated humanely and with regard for alleviation of pain in accordance with the *Guide for Care and Use of Laboratory Animals* (Institute of Laboratory Animal Resources 1996).

To study the response to various doses of E_2_, female mice (*n* = 5/dose for each strain) were ovariectomized on postnatal day (PND) 25 under isoflurane anesthesia, and Alzet osmotic pumps (Alza Corp., Palo Alto, CA) were implanted subcutaneously as previously described (Vandenberg et al. 2006). The pumps were filled with solutions delivering 0, 0.25, 0.5, 1, 2.5, 5, 10, or 50 μg E_2_/kg bw/day (Steraloids Inc., Newport, RI) dissolved in 50% dimethylsulfoxide (DMSO; vehicle control; Sigma-Aldrich, St. Louis, MO) for the length of treatment. The mice were killed on PND35, and their mammary glands and uteri were collected. For positive controls, we used the range of E_2_ doses previously reported in a mouse mammary gland assay by Skarda (2002) and a mouse uterotrophic assay (Padilla-Banks et al. 2001) based on the wet weight of the uterus (a standard for estrogen exposure).

To study the effects of perinatal exposure to BPA on responses to E_2_, male and female mice of each strain were paired. The morning on which a vaginal plug was observed was designated day 1 of pregnancy. On the evening of day 8 of pregnancy, dams were weighed and implanted subcutaneously with Alzet osmotic pumps releasing either vehicle (50% DMSO) or 250 ng BPA/kg bw/day (Sigma-Aldrich) dissolved in vehicle. For convenience, we refer to these groups as 0BPA and 250BPA, respectively. The pumps delivered BPA or vehicle until PND2. The offspring were culled to eight pups per dam on PND1. On PND25, three pups from each litter were ovariectomized, and pumps administering vehicle (50% DMSO) or 0.5 or 1μg E_2_/kg bw/day were implanted subcutaneously. These females were weighed and killed on PND35 (CD-1: *n* = 9–11/treatment group; C57Bl6: *n* = 6–7/treatment group).

### Tissue collection

The fourth inguinal mammary glands were dissected out; one mammary gland was immediately immersed in RNA*later* (Ambion Inc., Austin, TX) and stored at −80°C for RNA isolation. The second mammary gland was whole mounted as described previously (Markey et al. 2001a). Uteri were dissected, blotted on filter paper, and weighed.

### Morphometric analysis of mammary glands

Slides were coded and analysis was performed in a blind fashion. Mammary gland images were captured with a Zeiss dissection scope and AxioCam digital camera (Carl Zeiss Inc., Germany). Morphometric analysis was performed as described previously (Vandenberg et al. 2006). The parameters examined include number and area of terminal end buds (TEBs), area subtended by the ductal tree, and ductal extension measured as the distance from the midpoint of the lymph node to the leading edge of the ductal tree. In cases where the leading edge of the ductal tree did not grow beyond the center of the lymph node, ductal extension was given a negative value.

### RNA isolation and real-time quantitative RT-PCR

Mammary gland RNA was isolated and quantitative real time reverse transcription-polymerase chain reaction (RT-PCR) was performed as described previously (Munoz de Toro et al. 2005). The primers used were Msx2, forward: ggaagaccagatggaccaga, and reverse: tctgtatcaagtggccctgtc; amphiregulin, forward: aaggaggcttcgacaagaaa, and reverse: atccgaaagctccacttcct; and ribosomal protein L19 (a housekeeping gene), forward: atcgccaatgccaactcc, and reverse: tcatccttctcatccaggtca.

### Analysis of dose–response curves

For each parameter, the following were calculated: *a*) the lowest observable effect level (LOEL) dose (the lowest dose causing a statistically significant effect); *b*) the peak response [the dose(s) at which the response reaches a plateau or the highest response is achieved]; *c*) the half-maximal dose (the amount of E_2_ inducing half the maximal response); and *d*) the fold change (the peak response divided by the response of the ovariectomized control).

Curve-fitting analysis was performed for each parameter to determine whether the response to estradiol best conformed to a sigmoidal (monotonic) curve, or to a polynomial (inverted-U–shaped) curve. To determine which responses could be considered statistically nonmonotonic, we assessed whether the peak response of a given parameter occurred at one of the intermediate doses and whether the peak response could be statistically distinguished from the response at the highest dose. When both criteria were met, the parameter of interest was defined as having a nonmonotonic response to E_2_.

### Statistical analysis

Statistical significance was determined using SPSS software (SPSS, Chicago, IL). To determine statistical differences between the responses at each dose or the effects of BPA, we used nonparametric Mann-Whitney *U*-tests as well as parametric *t*-tests when data were normally distributed. To compare the responses of CD-1 and C57Bl6 mice, we used two-way analysis of variance (ANOVA). Post hoc tests (Bonferroni or planned *t*-tests) were used to make comparisons between groups. For all statistical tests, results were considered significant at *p* < 0.05. Values in figures are mean ± SE.

## Results

### Effect of E_2_ on uterine weight

**Figure 1:**
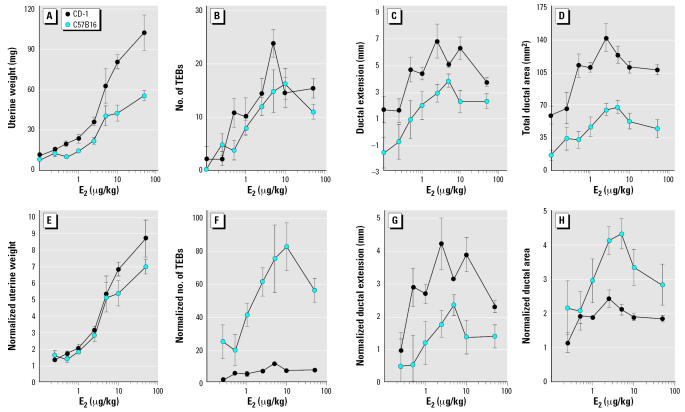
Estrogen dose–response curves (*A–D*) and the values for each parameter normalized to the ovariectomized control (*E–H*) for CD-1 and C57Bl6 mice. Uterine weight (*A, E*), number of TEBs (*B, F*), ductal extension (*C, G*), and ductal area (*D, H*). Values shown are mean ± SE.

Uterine wet weight represents the classical end point for assessing estrogenicity. As expected, the uterus demonstrated a monotonic dose–response curve to E_2_ in both mouse strains at the doses tested (Table 1, Figure 1A). We observed significant differences between strains at E_2_ doses ≥ 0.5 μg/kg bw/day. However, because CD-1 females are significantly larger than their C57B16 counterparts (25.5 ± 0.2 g and 16.3 ± 0.2 g, respectively; *p* < 0.001), significant differences between strains were not apparent when uterine weight was normalized to body weight (not shown). When normalized to the ovariectomized controls, the fold change in uterine weight was also comparable in both strains (Figure 1E).

### Morphometric parameters of the mammary gland: response to E_2_

In CD-1 females, E_2_ treatment increased the number and area of TEBs (Table 1, Figure 1B) and increased ductal extension (Figure 1C). Each of these parameters revealed an inverted-U–shaped, nonmonotonic response (Table 1). Graphic representation of the ductal area also showed an obvious inverted-U–shaped response to E_2_ (Figure 1D).

In C57Bl6 females, we found that the effect of E_2_ on the number of TEBs, area of TEBs, ductal area, and ductal extension generated an inverted-U–shaped dose response (Figure 1, Table 1). For every parameter, the doses showing peak responses were similar to those obtained in CD-1 females (Table 1). The LOEL for the number and area of TEBs occurred at a lower dose than in CD-1 females. However, for ductal area and ductal extension, the LOEL was found at a higher dose.

When CD-1 and C57Bl6 responses were compared, we detected no significant differences at any E_2_ dose for number or area of TEBs. However, significant differences were detected in parameters related to the overall growth of the epithelium (i.e., ductal extension and ductal area; Figure 1C–D). Because they were detected even in ovariectomized females (without E_2_ treatment), these differences appeared to be largely due to the divergent body size of these two strains. In fact, when normalized to body weight, strain differences only remained for ductal area; disparities between strains disappeared for the other parameters (not shown). Finally, 2-way ANOVAs did not indicate a significant interaction variable between E_2_ dose and mouse strain for any mammary gland parameter, indicating that the shape of the dose–response curves cannot be statistically distinguished for CD-1 and C57Bl6 strains.

The number of TEBs per ductal area (TEBs/area) and the area of all TEBs per ductal area (TEB area/area) were calculated to determine the TEB density. We found significant differences between these strains at 1, 10, and 50 μg E_2_/kg bw/day regarding both TEB density parameters (data not shown). At all three doses, the response observed in C57Bl6 females was more pronounced than that in CD-1 females. Because these parameters are ratios of the number or area of TEBs (no significant differences shown between strains) and ductal area (significant differences shown between strains, which correlate with animal size), the overall response to E_2_ may be more striking in the C57Bl6 strain because of their smaller size and/or slower development.

To explore this concept further, we normalized the number and area of TEBs, ductal area, and ductal extension to ovariectomized controls. The mammary glands of ovariectomized C57Bl6 mice displayed almost complete developmental arrest, whereas those of their CD-1 counterparts maintained a few TEB structures. Accordingly, E_2_ treatment increased the number of TEBs by 80-fold in C57Bl6 and only 10-fold in CD-1 mice (Figure 1F, Table 1), while the maximal TEB number was similar (Figure 1B). The increase in ductal extension was comparable in both strains (Figure 1G, Table 1), whereas the increase in ductal area was 1.4-fold in CD-1 and 3.3-fold in C57Bl6 mice (Figure 1H).

### Mammary gland gene expression: response to E_2_

**Figure 2:**
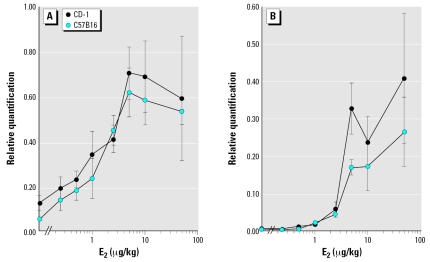
Gene expression in CD-1 and C57Bl6 mice. Expression of (*A*) Msx2 and (*B*) amphiregulin mRNA in the mammary glands was quantified using real-time RT-PCR. The values (mean ± SE) are fold change with respect to a calibrator sample.

The expression of Msx2 and amphiregulin mRNAs, two estrogen-regulated genes (Mallepell et al. 2006; Phippard et al. 1996), increased monotonically with increasing doses of E_2_ in both CD-1 and C57B16 mice (Figure 2 and Table 1).

### Selection of doses for pubertal E_2_ challenge

To investigate the effects of perinatal BPA exposure on the response to E_2_ at puberty, we used the dose–response curves of the morphometric points described above to identify two doses higher than the LOEL but lower than the half-maximal response for each strain. The half-maximal doses ranged from 0.40 to 2.0 μg E_2_/kg bw/day in CD-1 and 0.6 to 1.05 μg E_2_/kg bw/day in C57Bl6 mice (Table 1). From these calculations, we chose to administer an E_2_ challenge of 0, 0.5, or 1μg/kg bw/day.

### Morphometric analysis of the mammary gland: response to BPA

**Figure 3:**
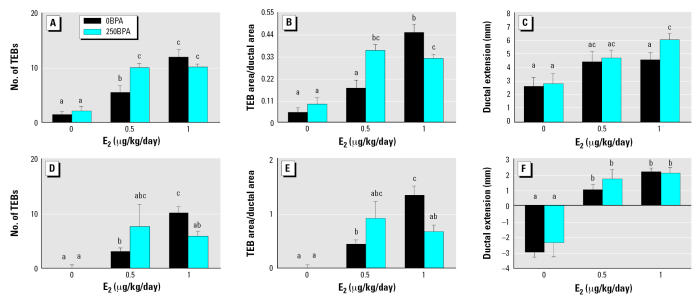
The effects of perinatal BPA exposure on an E_2_ challenge at puberty shown as the (*A*, *D*) number of TEBs, (*B*, *E*) TEB area/ductal area, and (*C*, *F*) ductal extension. There were no TEBs in the mammary glands of C57Bl6 E_2_ controls. (*A–C*) represent CD-1 mice and (*D–F*) represent C57Bl6 mice. Values shown are mean ± SE. Letters that are not in common indicate significant differences (*p* < 0.05).

In CD-1 females, the number of TEBs increased with higher doses of E_2_, as expected from the dose–response data. However, after administration of 0.5 μg E_2_/kg bw/day, the number of TEBs in 250BPA CD-1 mice increased significantly over that observed in 0BPA mice (Figure 3A). Other TEB parameters, such as the total area covered by TEBs (Table 2), TEBs/area (Table 2), and TEB area/ductal area (Figure 3B), were also significantly enhanced by this treatment. Additionally, the TEB area/area measured in the 250BPA group treated with 1 μg E_2_/kg bw/day was significantly lower (*p* < 0.015) than that measured in the 0BPA treated with the same dose of E_2_ (Figure 3B). When we measured ductal length, there was a significant increase in 250BPA challenged with 1 μg E_2_/kg bw/day compared with 0BPA mice (Figure 3C). Additionally, increasing doses of E_2_ administered to CD-1 mice induced overall growth of the ductal epithelium, measured as increased ductal area. However, perinatal exposure to BPA had no effect on this parameter (Table 2).

In C57Bl6 mice, the number of TEBs increased with higher doses of E_2_, as expected from the dose–response data. In 250BPA females, the mean number of TEBs induced by 0.5 μg E_2_/kg bw/day was increased compared with 0BPA animals (Figure 3D), similar to the response seen in the CD-1 strain; however, these differences were not statistically significant, likely due to variability in the data. Additionally, treatment with 1 μg E_2_/kg bw/day induced significantly fewer TEBs in 250BPA compared with 0BPA females (Figure 3D). The total TEB area induced by administration of 1 μg E_2_/kg bw/day was lower in the 250BPA mice compared with 0BPA animals (Table 2), although this decrease was not statistically significant. As in CD-1 mice, TEB parameters such as TEBs/area (Table 2) and TEB area/area (Figure 3E) were significantly lower in the 250BPA mice treated with 1μg E_2_/kg bw/day compared with 0BPA in C57Bl6 mice.

Parameters associated with overall ductal growth (ductal area and extension) were also measured in the C57Bl6 mammary gland. As expected, higher doses of E_2_ induced larger areas (Table 2) and greater ductal extensions (Figure 3F) than in ovariectomized controls. As observed in CD-1 mice, perinatal exposure to BPA did not alter ductal area; BPA also did not alter ductal extension in C57Bl6, contrasting with growth patterns observed in CD-1 mice.

### Uterine weight: response to BPA

As expected, treatment with increasing doses of E_2_ resulted in increased uterine wet weight. In both strains, perinatal treatment with 250 ng BPA/kg bw/day did not alter this parameter (Table 2).

## Discussion

In the present study we examined two important issues regarding estrogen action—strain sensitivity and dose–response curve shape—always in the context of the effects of perinatal BPA exposure. Previously, we reported that perinatal exposure to environmentally relevant levels of BPA results in altered postnatal development of the mammary gland (Markey et al. 2001a, 2003a; Munoz de Toro et al. 2005). One of the most striking observations was that the sensitivity of the mammary gland to E_2_ increased in perinatally BPA-exposed CD-1 females that were ovariectomized before puberty (Munoz de Toro et al. 2005). The present study revealed that both outbred CD-1 and inbred C57Bl6 strains respond quite similarly to E_2_ and BPA in many parameters. The C57Bl6 strain is of particular interest because it is widely used to study mammary gland development and in the generation of genetically modified mice.

There are concerns regarding the use of laboratory strains selected for large litter size (i.e., CD-1 mice) stemming from the possible correlation with resistance to endocrine disruptors including xenoestrogens. It has been suggested that testing chemicals in these strains may underestimate the endocrine disruptor potential of the agent being examined (Spearow et al. 1999). Specifically, CD-1 males were shown to be E_2_-resistant when compared with C57Bl6 mice regarding effects on testes weight and spermatogenesis (Spearow et al. 1999). Strain susceptibility was also observed in rats, often specific to the chemical being studied. For example, Sprague-Dawley rats were resistant to BPA compared with the Fischer 344 rats regarding proliferation of vaginal epithelium. However, both strains responded equally to E_2_. Thus, the differences between Sprague-Dawley and Fischer 344 rats suggest that there may be strain-specific differences that affect the metabolism of a particular estrogenic chemical or class of chemicals (Long et al. 2000).

The shape of the dose–response curve should also be considered when assessing strain sensitivity. When a given parameter exhibits a monotonic dose–response curve, all effective doses should result in a qualitatively similar effect. To the contrary, if the dose–response curve is nonmonotonic or has an inverted-U shape, opposite effects might be observed at different doses. It has been proposed that non-monotonic dose–response curves are generated by the integration of two or more monotonic dose–response curves that are occurring through different pathways and affecting a common end point with opposing effects (Conolly and Lutz 2004). For those end points, one cannot test a single high dose of a given chemical to assess whether or not it produces a biological effect (vom Saal and Hughes 2005; Welshons et al. 2003). In the present study, morphometric parameters of the mammary glands of both CD-1 and C57Bl6 mice displayed inverted-U–shaped dose–response curves to E_2_. Although most parameters in CD-1 mice met the criteria for statistical nonmonotonicity, variability in responses prevented the C57Bl6 from meeting this standard. One striking difference between these strains was the state of quiescence of the mammary gland of C57Bl6 ovariectomized mice compared with their CD-1 counterparts. There were practically no TEBs in the former, whereas a few TEBs were present in the CD-1 females 10 days after ovariectomy. It is possible that these results are indicative of different rates of development or onset of puberty in females of these two strains. Although there is no evidence in the literature to support this conclusion, no studies have examined age of puberty in these two strains under the same conditions (food supplied, light cycle, temperature, etc.) In the present study, females were ovariectomized before puberty, and thus this information could not be collected.

In contrast to the morphometric parameters, the induction of estrogen-target genes in the mammary gland was monotonic in both strains. The magnitude of the response was comparable in the two strains, and the sensitivities of these responses were lower than those of several morphological end points.

### E_2_ sensitivity of the uterus

Diel et al. (2004) found that the uterus and vagina of several rat strains responded similarly to three different doses of estrogen in a 3-day assay (Diel et al. 2004). In the present study, we arrived at a similar conclusion using a set of seven different E_2_ doses and a 10-day assay in mice. Both mouse strains displayed a monotonic dose–response curve regarding uterine weight at the doses tested, and their response was comparable when uterine weight was normalized to body weight. Similar dose–response curves were reported in a 3-day (Padilla-Banks et al. 2001) and a 10-day mouse assay (Skarda 2002).

Overall, this study revealed little or no difference in the sensitivity to E_2_ between CD-1 and C57Bl6 mice regarding the uterotrophic response and a variety of morphometric and gene-expression end points in the mammary gland. This is in contrast to the marked differences observed between these strains in testis end points (Spearow et al. 1999). The mechanisms underlying the latter differences have yet to be determined. However, steroid metabolism by the liver is subject to hormonal imprinting (Gustafsson et al. 1977), which may explain differences in response between males and females of the same strain.

### Effect of perinatal exposure to BPA on the pubertal response to E_2_

Perinatal BPA exposure did not alter the uterine response to E_2_ administered from PND25 to PND35 in either mouse strain. In contrast, the response of the mammary gland to E_2_ was significantly altered by perinatal BPA exposure. A pattern emerged for TEB-related parameters, which increased in response to 0.5 μg E_2_/kg bw/day relative to controls, suggesting a shift to the left of the dose–response curve. Increased responses at 0.5 μg E_2_/kg bw/day were significant in CD-1 mice but did not reach significance in C57Bl6. The increase in number of TEBs induced by 1 μg E_2_/kg bw/day was significantly reduced in the BPA-pretreated C57Bl6 mice. These results suggest a difference between the two strains in the level of the response to E_2_ after perinatal BPA exposure, such that increased sensitivity to E_2_ was manifested at a lower dose in CD-1 than in C57Bl6 mice. Alternatively, the sensitivity of the two strains may be similar, but the higher variability in the C57Bl6 TEB-related parameters may have precluded reaching statistical significance for end points that were significantly altered in the CD-1 strain. However, in both strains perinatal exposure to BPA significantly altered the response to E_2_ later in life.

Estrogen exposure represents a main risk factor for breast carcinogenesis. Increased sensitivity to E_2_ may have similar effects. Consistent with this concept, previous studies of CD-1 mice exposed perinatally to BPA revealed an increased number of TEBs at puberty and terminal ends in adulthood (Markey et al. 2001a; Munoz de Toro et al. 2005). These two structures are thought to be the sites where neoplasias arise. Also, there is an overexpression of progesterone receptors in BPA-exposed animals, which in turn induces excessive lateral branching of the mammary gland ducts (Munoz de Toro et al. 2005), resulting in an increased ductal density of the gland. In humans, increased mammographic density is also a risk factor for breast cancer (McCormack and dos Santos Silva 2006). In the present study we extend these findings to C57Bl6 mice, suggesting that the enhanced sensitivity to E_2_ resulting from perinatal BPA exposure may represent a general phenomenon in mice rather than a strain idiosyncrasy.

### Nonmonotonic dose–response curves

*In vitro* studies that used human breast epithelial cells and other estrogen-target cell lines showed nonmonotonic dose–response curves in response to increasing E_2_ doses (Amara and Dannies 1983; Soto and Sonnenschein 1985). This type of curve suggests that estrogens can evoke different effects, such as induction (Soto and Sonnenschein 1987) or inhibition of cell proliferation (Szelei et al. 2000), depending on the dose tested. The combined effect of these variable responses is reflected in the overall cell number (Soto and Sonnenschein 2001). In the mammary gland, estrogens promote proliferation—manifested as ductal growth (Nandi 1958)—and induce apoptosis—manifested as lumen formation (Munoz de Toro et al. 2005).

## Conclusions

Nonmonotonic dose–response curves are observed in cultured cells and in animal models, and are oftentimes observed for endocrine end points. These patterns highlight the unreliability of assuming that the effect of exposure to low doses of a hormone, endocrine disruptor, or other toxicant can be extrapolated from the response to high doses of the compound (Conolly and Lutz 2004; vom Saal and Hughes 2005).

Contrary to the clear differences in the testicular response to postnatal administration of E_2_ between CD-1 and C57Bl6 mice (Spearow et al. 1999), the differences observed in the uterus and mammary gland of these different mouse strains are subtle. In addition, the mammary glands of both strains are sensitive to perinatal exposure to low doses of BPA, in that the postnatal response to E_2_ is significantly modified. This observation suggests that both strains provide adequate models for the study of perinatal exposure to xenoestrogens. Even though the outbred CD-1 strain has been selected for large litter size and reproductive efficiency, these results show that this strain provides an excellent model for the study of estrogen action in the uterus and mammary gland. Additionally, mice of the C57Bl6 strain may be used advantageously in the study of endocrine disruption when an inbred strain is required. Confirmation of the suitability of the C57Bl6 strain for this work is important because many genetically modified animals have been developed on this background.

## Figures and Tables

**Table 1:** Dose–response curves for uterine wet weight and mammary gland morphological parameters in both mouse strains.

	CD-1 Mice					C57Bl6 mice				
Parameter	LOEL	Half-max dose	Dose of max effect	Fold change	Curve shape	LOEL	Half-max dose	Dose of max effect	Fold change	Curve shape
Ductal extension	0.5	0.42	2.5–10	3.2	Inverted-U	2.5	0.6	1–50	−3.34	Inverted-U
No. of TEBs	2.5	1.8	5	9.8	Inverted-U	0.5	1.05	2.5–50	80.25	Inverted-U
TEB area	2.5	1.6	5	11.6	Inverted-U	1	1	2.5–50	97.44	Inverted-U
Ductal area	0.5	0.4	1–50	1.4	Inverted-U	2.5	0.78	1–50	3.3	Inverted-U
No. of TEBs/ductal area	2.5	2	5–10	7.0	—a	0.5	0.85	5–50	62.25	—a
TEB area/ductal area	2.5	1.6	2.5–10	8.6	—a	0.5	0.8	5–50	75.96	—a
Uterine weight	0.25	4.25	10–50	4.7	Monotonic	1.0	3.55	5–50	5.95	Monotonic
Uterine weight/bw	0.25	4.1	10–50	7.8	Monotonic	1.0	3.4	5–50	6.09	Monotonic
Msx2	2.5	2.5	5–50	4.7	Monotonic	0.5	1.5	5–50	10.6	Monotonic
Amphiregulin	1	3.6	5–50	64.8	Monotonic	2.5	4	5–50	81.81	Monotonic

max, maximal. The LOEL, half-maximal dose, dose of maximal effect, fold change, and curve shape were calculated.

a The curve shape for these parameters is “derived” data, from a quotient between two direct measurements, each one of them being affected by E_2_; therefore, the shape of the derived curve is irrelevant.

**Table 2:** Mammary gland morphological parameters of CD-1 and C57Bl6 mice exposed perinatally to BPA (0BPA and 250BPA) and postnatally to 0, 0.5, or 1 μg E_2_/kg bw/day.

	E_2_ (μg/kg bw/day), BPA					
Treatment	0, 0BPA	0, 250BPA	0.5, 0BPA	0.5, 250BPA	1, 0BPA	1, 250BPA
CD-1 mice						
Ductal extension	2.62 ± 0.63^a^	2.80 ± 0.76^a^	4.41 ± 0.74^a,b^	4.69 ± 0.58^a,b^	4.54 ± 0.53^a^	6.00 ± 0.46^b^
# TEBs	1.4 ± 0.58^a^	2.1 ± 0.78^a^	5.5 ± 1.2^b^	10.0 ± 0.65^c^	11.9 ± 1.3^c^	10.1 ± 0.50^c^
TEB area	0.048 ± 0.020^a^	0.078 ± 0.029^a^	0.219 ± 0.054^b^	0.428 ± 0.040^d^	0.546 ± 0.049^c^	0.440 ± 0.034^c,d^
Ductal area	79.1 ± 8.7^a^	82.26 ± 7.0^a^	126.1 ± 9.2^b^	118.6 ± 8.2^b^	124.58 ± 8.65^b^	139.8 ± 6.8^b^
# TEBs/ductal area	0.015 ± 0.006^a^	0.023 ± 0.008^a^	0.042 ± 0.009^a^	0.085 ± 0.006^b^	0.096 ± 0.009^b^	0.075 ± 0.005^b^
TEB area/ductal area	0.0005 ± 0.0002^a^	0.0009 ± 0.0003^a^	0.0017 ± 0.0004^a^	0.0036 ± 0.0003^b,c^	0.0045 ± 0.0004^b^	0.0032 ± 0.0002^c^
Uterine weight	15.32 ± 1.58^a^	14.97 ± 0.73^a^	20.59 ± 1.02^b^	19.46 ± 0.91^b^	25.47 ± 1.46^b,c^	28.32 ± 1.33^c^
C57Bl6 mice						
Ductal extension	−3.00 ± 0.34^a^	−2.39 ± 0.93^a^	0.99 ± 0.34^b^	1.68 ± 0.59^b^	2.13 ± 0.25^b^	2.05 ± 0.36^b^
# TEBs	0.0 ± 0.0^a^	0.0 ± 0.0^a^	3.1 ± 0.6^b^	7.7 ± 4.0^a,b,c^	10.1 ± 1.1^c^	6.0 ± 0.8^a,b^
TEB area	0.00 ± 0.00^a^	0.00 ± 0.00^a^	14.57 ± 2.72^a^	36.83 ± 16.21^a^	54.14 ± 8.23^b^	30.50 ± 5.98^a,b^
Ductal area	6.40 ± 0.80^a^	10.10 ± 2.64^a^	29.13 ± 1.33^b^	36.03 ± 4.49^b,c^	40.58 ± 3.41^c^	44.69 ± 2.06^c^
# TEBs/ductal area	0.00 ± 0.00^a^	0.00 ± 0.00^a^	0.11 ± 0.019^b^	0.18 ± 0.072^a,b,c^	0.25 ± 0.018^c^	0.13 ± 0.015^a,b^
TEB area/ductal area	0.00 ± 0.00^a^	0.00 ± 0.00^a^	0.44 ± 0.080^b^	0.91 ± 0.31^a,b,c^	1.34 ± 0.16^c^	0.67 ± 0.12^a,b^
Uterine weight	5.1 ± 0.26 ^a^	8.05 ± 2.6^a,b^	9.31 ± 0.71^b^	11.00 ± 1.58^b,c^	12.20 ± 0.76^c^	13.0 ± 1.53^c^

Letters that are not in common indicate significant differences (*p* < 0.05).

## References

[b1-ehp0115-000592] Alworth LC, Howdeshell KL, Ruhlen RL, Day JK, Lubahn DB, Huang TH-M (2002). Uterine responsiveness to estradiol and DNA methylation are altered by fetal exposure to diethylstilbestrol and methoxychlor in CD-1 mice: effects of low versus high doses. Toxicol Appl Pharmacol.

[b2-ehp0115-000592] Amara JF, Dannies PS (1983). 17 β-Estradiol has a biphasic effect on GH cell growth. Endocrinology.

[b3-ehp0115-000592] Arakawa C, Fujimaki K, Yoshinaga J, Imai H, Serizawa S, Shiraishi H (2004). Daily urinary excretion of bisphenol A. Environ Health Prev Med.

[b4-ehp0115-000592] Biles JE, McNeal TP, Begley TH, Hollifield HC (1997). Determination of bisphenol-A in reusable polycarbonate food-contact plastics and migration to food simulating liquids. J Agric Food Chem.

[b5-ehp0115-000592] Brotons JA, Olea-Serrano MF, Villalobos M, Olea N (1994). Xenoestrogens released from lacquer coating in food cans. Environ Health Perspect.

[b6-ehp0115-000592] Calafat AM, Kuklenyik Z, Reidy JA, Caudill SP, Ekong J, Needham LL (2005). Urinary concentrations of bisphenol A and 4-nonylphenol in a human reference population. Environ Health Perspect.

[b7-ehp0115-000592] Conolly RB, Lutz WK (2004). Nonmonotonic dose-response relationships: mechanistic basis, kinetic modeling, and implications for risk assessment. Toxicol Sci.

[b8-ehp0115-000592] Davis DL, Bradlow HL, Wolff M, Woodruff T, Hoel DG, Anton-Culver H (1993). Medical hypothesis: xenoestrogens as preventable causes of breast cancer. Environ Health Perspect.

[b9-ehp0115-000592] Diel P, Schmidt S, Vollmer G, Janning P, Upmeier A, Michna H (2004). Comparative responses of three rat strains (DA/Han, Sprague-Dawley and Wistar) to treatment with environmental estrogens. Arch Toxicol.

[b10-ehp0115-000592] Endocrine Disruptor Screening and Testing Advisory Committee. 1998. Endocrine Disruptor Screening and Testing Advisory Committee Final Report 2006. Available: http://www.epa.gov/scipoly/oscpendo/edspoverview/finalrpt.htm [accessed 28 November 2006].

[b11-ehp0115-000592] Gustafsson JA, Eneroth P, Pousette A, Skett P, Sonnenschein C, Stenberg A (1977). Programming and differentiation of rat liver enzymes. J Steroid Biochem.

[b12-ehp0115-000592] Ikezuki Y, Tsutsumi O, Takai Y, Kamei Y, Taketani Y (2002). Determination of bisphenol A concentrations in human biological fluids reveals significant early prenatal exposure. Hum Reprod.

[b13-ehp0115-000592] Institute of Laboratory Animal Resources 1996. Guide to Care and Use of Laboratory Animals. Washington, DC:National Academy Press.

[b14-ehp0115-000592] Long X, Steinmetz R, Ben-Jonathan N, Caperell-Grant A, Young PC, Nephew KP (2000). Strain differences in vaginal responses to the xenoestrogen bisphenol A. Environ Health Perspect.

[b15-ehp0115-000592] Mallepell S, Krust A, Chambon P, Brisken C (2006). Paracrine signaling through the epithelial estrogen receptor α is required for proliferation and morphogenesis in the mammary gland. Proc Natl Acad Sci USA.

[b16-ehp0115-000592] Markey CM, Coombs MA, Sonnenschein C, Soto AM (2003a). Mammalian development in a changing environment: exposure to endocrine disruptors reveals the developmental plasticity of steroid-hormone target organs. Evol Dev.

[b17-ehp0115-000592] Markey CM, Luque EH, de Toro MM Munoz, Sonnenschein C, Soto AM (2001a). *In utero* exposure to bisphenol A alters the development and tissue organization of the mouse mammary gland. Biol Reprod.

[b18-ehp0115-000592] Markey CM Michaelson CL Sonnenschein C Soto AM 2001b. Alkylphenols and bisphenol A as environmental estrogens. In: The Handbook of Environmental Chemistry Vol 3. Part L, Endocrine Disruptors - Part I (Metzler M, ed). Berlin/Heidelberg:Springer-Verlag, 129–153.

[b19-ehp0115-000592] Markey CM, Rubin BS, Soto AM, Sonnenschein C (2003b). Endocrine disruptors from Wingspread to environmental developmental biology. J Steroid Biochem Molec Biol.

[b20-ehp0115-000592] McCormack VA, dos Santos Silva I (2006). Breast density and parenchymal patterns as markers of breast cancer risk: a meta-analysis. Cancer Epidemiol Biomarkers.

[b21-ehp0115-000592] de Toro MM Munoz, Markey CM, Wadia PR, Luque EH, Rubin BS, Sonnenschein C (2005). Perinatal exposure to bisphenol A alters peripubertal mammary gland development in mice. Endocrinology.

[b22-ehp0115-000592] Nandi S (1958). Endocrine control of mammary gland development and function in the C3H/He Crgl mouse. J Natl Cancer Inst.

[b23-ehp0115-000592] NTP 2001. National Toxicology Program’s Report of the Endocrine Disruptors Low-Dose Peer Review. Research Triangle Park, NC:National Toxicology Program. Available: http://ntp.niehs.nih.gov/ntp/htdocs/liason/LowDosePeerFinalRpt.pdf [accessed 5 March 2006].

[b24-ehp0115-000592] Olea N, Pulgar R, Perez P, Olea-Serrano F, Rivas A, Novillo-Fertrell A (1996). Estrogenicity of resin-based composites and sealants used in dentistry. Environ Health Perspect.

[b25-ehp0115-000592] Padilla-Banks E, Jefferson WN, Newbold RR (2001). The immature mouse is a suitable model for detection of estrogenicity in the uterotropic bioassay. Environ Health Perspect.

[b26-ehp0115-000592] Phippard DJ, Weber-Hall SJ, Sharpe PT, Naylor MS, Jayatalake H, Maas R (1996). Regulation of Msx-1, Msx-2, Bmp-2 and Bmp-4 during foetal and postnatal mammary gland development. Development.

[b27-ehp0115-000592] Rubin BS, Murray MK, Damassa DA, King JC, Soto AM (2001). Perinatal exposure to low doses of bisphenol A affects body weight, patterns of estrous cyclicity, and plasma LH levels. Environ Health Perspect.

[b28-ehp0115-000592] Schonfelder G, Wittfoht W, Hopp H, Talsness CE, Paul M, Chahoud I (2002). Parent bisphenol A accumulation in the human maternal-fetal-placental unit. Environ Health Perspect.

[b29-ehp0115-000592] Sharpe RM, Skakkebaek NE (1993). Are oestrogens involved in falling sperm count and disorders of the male reproductive tract?. Lancet.

[b30-ehp0115-000592] Skakkebaek NE, Meyts ER, Jorgensen N, Carlsen E, Petersen PM, Giwercman A (1998). Germ cell cancer and disorders of spermatogenesis: an environmental connection?. APMIS.

[b31-ehp0115-000592] Skarda J (2002). Sensitivity and specificity of bioassay of estrogenicity on mammary gland and uterus of female mice. Physiol Res.

[b32-ehp0115-000592] Soto AM Lin T-M Justicia H Silvia RM Sonnenschein C 1992. An “in culture” bioassay to assess the estrogenicity of xenobiotics. In: Chemically-Induced Alterations in Sexual Development: The Wildlife/human Connection (Colborn T, Clement C, eds). Princeton, NJ:Princeton Scientific Publishing, 295–309.

[b33-ehp0115-000592] Soto AM, Sonnenschein C (1985). The role of estrogens on the proliferation of human breast tumor cells (MCF-7). J Steroid Biochem.

[b34-ehp0115-000592] Soto AM, Sonnenschein C (1987). Cell proliferation of estrogen-sensitive cells: the case for negative control. Endocr Rev.

[b35-ehp0115-000592] Soto AM, Sonnenschein C (2001). The two faces of Janus: sex steroids as mediators of both cell proliferation and cell death. J Natl Cancer Inst.

[b36-ehp0115-000592] Spearow JL, Doemeny P, Sara R, Leffler R, Barkley M (1999). Genetic variation in susceptibility to endocrine disruption by estrogen in mice. Science.

[b37-ehp0115-000592] Szelei J, Soto AM, Geck P, Desronvil M, Prechtl NV, Weill BC (2000). Identification of human estrogen-inducible transcripts that potentially mediate the apoptotic response in breast cancer. J Steroid Biochem Mol Biol.

[b38-ehp0115-000592] Takeuchi T, Tsutsumi O (2002). Serum bisphenol A concentrations showed gender differences, possibly linked to androgen levels. Biochem Biophys Res Commun.

[b39-ehp0115-000592] Vandenberg LN, Wadia PR, Schaeberle CM, Rubin BS, Sonnenschein C, Soto AM (2006). The mammary gland response to estradiol: monotonic at the cellular level, non-monotonic at the tissue-level of organization?. J Steroid Biochem Mol Biol.

[b40-ehp0115-000592] vom Saal FS, Hughes C (2005). An extensive new literature concerning low-dose effects of bisphenol A shows the need for a new risk assessment. Environ Health Perspect.

[b41-ehp0115-000592] vom Saal FS, Timms BG, Montano MM, Palanza P, Thayer KA, Nagel SC (1997). Prostate enlargement in mice due to fetal exposure to low doses of estradiol or diethylstilbestrol and opposite effects at high doses. Proc Natl Acad Sci USA.

[b42-ehp0115-000592] Welshons WV, Thayer KA, Judy BM, Taylor JA, Curran EM, vom Saal FS (2003). Large effects from small exposures. I. Mechanisms for endocrine-disrupting chemicals with estrogenic activity. Environ Health Perspect.

